# A System of Medicine

**Published:** 1909-12

**Authors:** 


**A System of Medicine.** By Many Writers. Edited by Sir Clifford Allbutt, K.C.B., and Humphry Davy Rolleston, M.D. Vol. V. Pp. xii, 969. London: Macmillan & Co. Ltd. 1909.

Eleven years have passed since the corresponding volume of Allbutt's *System* appeared in its first edition, and the progress in medicine is made clear by the many changes necessary in writing of *Diseases of the Respiratory System and Disorders of the Blood.*

Acute Lobular Pneumonia and Broncho-pneumonia form a new chapter, in which Beddard and Eyre discuss the many infections which may be present to account for the several maladies included in the title.

Pulmonary Tuberculosis covers vast new fields. In the former edition Dr. Kidd alluded briefly to continental health resorts, adding, "The majority of English patients find such a rigid supervision irksome and disagreeable, and hitherto such establishments have not been in much request in this country."

In the second edition Sanatorium Treatment occupies eight pages, and is admirably described by Dr. Bardswell. The bacteriology of phthisis and cognate problems of immunity, including tuberculin treatment and determination of agglutinins or opsonins, loom large, and mark a huge step forward.

In blood diseases there is much that is new and much that is altered. Especially striking is the tendency to less dogmatism, and there are many relegations of diseases which formerly ranked as separate entities to the category of symptoms, or at best symptom-complexes.

Nowhere more than in hæmatology is Sir Thomas Browne's dictum applicable: " We do but learn to-day that which our better advanced judgements will unteach to-morrow."

